# A zebrafish HCT116 xenograft model to predict anandamide outcomes on colorectal cancer

**DOI:** 10.1038/s41419-022-05523-z

**Published:** 2022-12-23

**Authors:** Francesca Maradonna, Camilla M. Fontana, Fiorenza Sella, Christian Giommi, Nicola Facchinello, Chiara Rampazzo, Micol Caichiolo, Seyed Hossein Hoseinifar, Luisa Dalla Valle, Hien Van Doan, Oliana Carnevali

**Affiliations:** 1 Department of Life and Environmental Sciences, Polytechnic University of Marche, Ancona, Italy; 2 Biostructures and Biosystems National Institute, (INBB), Rome, Italy; 3 Department of Animal and Aquatic Sciences, Faculty of Agriculture, Chiang Mai University, Chiang Mai, Thailand; 4 National Research Council (CNR), Neuroscience Institute, Padova, Italy; 5 Department of Molecular Medicine, University of Padova, Padova, Italy; 6 Department of Biology, University of Padova, Padova, Italy; 7 Department of Fisheries, Faculty of Fisheries and Environmental Sciences, Gorgan University of Agricultural Sciences and Natural Resources, Gorgan, Iran

**Keywords:** Checkpoint signalling, Cancer models

## Abstract

Colon cancer is one of the leading causes of death worldwide. In recent years, cannabinoids have been extensively studied for their potential anticancer effects and symptom management. Several in vitro studies reported anandamide’s (AEA) ability to block cancer cell proliferation and migration, but evidence from in vivo studies is still lacking. Thus, in this study, the effects of AEA exposure in zebrafish embryos transplanted with HCT116 cells were evaluated. Totally, 48 hpf xenografts were exposed to 10 nM AEA, 10 nM AM251, one of the cannabinoid 1 receptor (CB1) antagonist/inverse agonists, and to AEA + AM251, to verify the specific effect of AEA treatment. AEA efficacy was evaluated by confocal microscopy, which demonstrated that these xenografts presented a smaller tumor size, reduced tumor angiogenesis, and lacked micrometastasis formation. To gain deeper evidence into AEA action, microscopic observations were completed by molecular analyses. RNA seq performed on zebrafish transcriptome reported the downregulation of genes involved in cell proliferation, angiogenesis, and the immune system. Conversely, HCT116 cell transcripts resulted not affected by AEA treatment. In vitro HCT116 culture, in fact, confirmed that AEA exposure did not affect cell proliferation and viability, thus suggesting that the reduced tumor size mainly depends on direct effects on the fish rather than on the transplanted cancer cells. AEA reduced cell proliferation and tumor angiogenesis, as suggested by *socs3* and *pcnp* mRNAs and Vegfc protein levels, and exerted anti-inflammatory activity, as indicated by the reduction of *il-11a*, *mhc1uba*, and *csf3b* mRNA. Of note, are the results obtained in groups exposed to AM251, which presence nullifies AEA’s beneficial effects. In conclusion, this study promotes the efficacy of AEA in personalized cancer therapy, as suggested by its ability to drive tumor growth and metastasis, and strongly supports the use of zebrafish xenograft as an emerging model platform for cancer studies.

## Introduction

Over the last decades, cannabinoids have been extensively used in palliative care to alleviate pain, relieve nausea and stimulate appetite in cancer patients [1]. However, several studies so far emphasized the importance of safety measures when using cannabinoids to avoid cognitive function impairment [2]. On the other hand, the role of cannabinoid-based drugs in the modulation of oxidative stress processes that underpin cognitive impairments emerged and recognized the endocannabinoid system (ECS) as the leading player in this neuroprotective activity [3]. Nevertheless, in the last years, most of the interest pointed toward increasing the knowledge on the antitumoral activity of cannabinoids, which depends on their capacity to interact with ECS networks or other cellular pathways, affecting the development/progression of diseases [4, 5]. Specifically, an increasing number of reports highlighted the role of cannabinoids in cancer spreading, invasion, angiogenesis, migration, and metastatic mechanism [6–8]. ECS agonists, by binding to cannabinoid receptors, can activate a Gi/o protein-coupled receptor that triggers a signal cascade responsible for the down-regulation of the proangiogenic factors, including vascular endothelial growth factors (VEGFs) and angiopoietin-2 (Ang-2) [7]. Moreover, cannabinoids inhibit angiogenesis and invasion, inducing the release of tissue inhibitors of matrix metalloproteinases-1 (TIMP-1) that, in turn, acts as an endogenous inhibitor of matrix metalloproteinase 2 (MMP2) [7]. Moreover, evidence regarding cannabinoid ability to affect the Wnt pathway, thus altering the epithelial-mesenchymal transition and chemoresistance, has been shown in different tumor types and has been associated with a reduction of β-catenin target genes and mesenchymal markers [9]. Focusing on N-arachidonoyl-ethanolamine, anandamide (AEA), the cannabinoid used in this study, previous in vitro results demonstrated its role in the inhibition of breast tumor-induced angiogenesis [10] and the alteration of metabolism, glycosylation profile, and migration of metastatic melanoma cells [11].

Starting from this scenario, this study aims at investigating the antitumoral effects of AEA on HCT116 cells, a human colorectal carcinoma cell line, and gaining insight into the mechanisms of action in vivo using a zebrafish xenograft model [12, 13]. Differently from patient-derived xenografts (PDX) and organoids which have significant disadvantages, including long growth times that introduce genetic and epigenetic changes to the tumor, the zebrafish xenograft assay offers several advantages [14, 15]. Young embryos lack an efficient immune system and therefore, the injected cancer cells are not rejected, and the formation of primary tumors and micrometastases is rapid. In addition, imaging of the small, transparent xenograft allows catching macroscopic changes regarding AEA effects either on the HCT116 transplanted cell or on the fish itself. All this macroscopic evidence will be flanked by a deeper investigation using molecular tools, e.g RNA seq and Western blot, which will provide bricks to understand the mechanism of action of AEA on tumoral cell proliferation and hopefully will be the starting point for future studies that will consider the use of this cannabinoid for in vivo cancer therapy.

## Results

### Effects of experimental treatments on tumor growth in HCT116 zebrafish xenografts

**Fig. 1:**
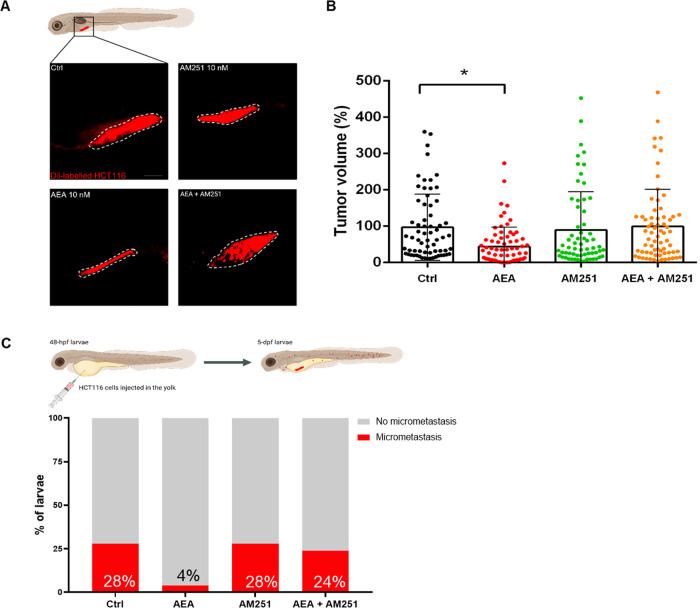
AEA exposure controls tumor size and metastasis onset in xenografts. **A** Representative confocal z-stack images of 5 dpf (3 dpi) larvae with tumor cells marked by the red fluorescence (Dil Vibrant red staining). Scale bar 100 µm. **B** Quantification of total Dil Vibrant Red fluorescence. The mean value of the tumor size in Ctrl was settled as 100%. The error bar indicates SEM. **C** Percentage of larvae presenting micrometastasis in the four experimental conditions. (Ctrl *n* = 66; AEA *n* = 73; AM251 *n* = 63; AEA + AM251 *n* = 69 from five independent experiments). Statistical analysis was performed using one-way ANOVA. *p < 0.01. Image created with Biorender.com.

The tumor volume was measured by confocal microscopy evaluating HCT116 cell fluorescence. Interestingly, in xenograft exposed to AEA, the tumor size resulted significantly smaller than in Ctrl (Fig. 1A, B). This result suggested that AEA can affect tumor growth by influencing normal cell proliferation. This hypothesis was further supported by evidence obtained from xenografts exposed either to 1-(2,4-dichlorophenyl)-5-(4-iodophenyl)-4-methyl-N-1-piperidinyl-1H-pyrazole-3-carboxamide (AM251), a cannabinoid 1 (CB1) receptor 1 inverse agonist/antagonist [16]/, or AEA + AM251, in which tumor size was similar to that of Ctrl larvae (Fig. 1B). Metastatic deposits, one of the main issues in cancer therapy, were further investigated in xenografts. HCT116 cell micro-metastasis were observed in all experimental groups, but in fish exposed to AEA, a significant reduction was recorded (4%) with respect to Ctrl fish (28%), suggesting the positive role of AEA against this pathological process. A similar percentage of fish presenting metastasis was observed among Ctrl (28%), AM251 (28%), and AEA + AM251 (24%) experimental groups (Fig. 1C).

### Effects of experimental treatments on tumor angiogenesis

**Fig. 2:**
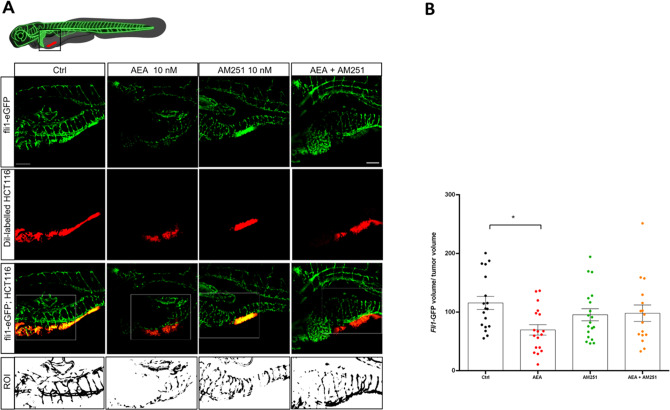
AEA reduces vascularization in *fli1*-GFP positive xenografts. **A** Representative confocal z-stack images of 5 dpf (3 dpi) larvae with the tumor highlighted by the red fluorescence (Dil Vibrant red staining) and blood vessels marked by the green fluorescence (Tg(*fli1*:EGFP)^y1^ line). Scale bar 100 µm. The white box indicates the region of interest (ROI) selected to quantify the vascularization. **B** Quantification of total green fluorescence of the ROI analyzed with Fiji. The mean value in the Ctrl was settled as 100%. The error bar indicates SEM. Statistical analysis was performed using one-way ANOVA. *p < 0.05; (Ctrl *n* = 18; AEA *n* = 18; AM251 *n* = 19; AEA + AM251 *n* = 16 from three independent experiments). Image created with Biorender.com.

The use of *Tg(fli1:EGFP)*^*y1*^ transgenic xenografts allows the visualization of fish vascularization (Fig. 2A). In particular, in these xenografts, a focus was made on blood vessels close to the tumor area, directly involved in its support and growth. In fish exposed to AEA, angiogenesis around the tumor (Fig. 2B) resulted less developed than in the other experimental groups. In particular, the intensity of the signal associated with blood vessels that generate from the subintestinal area was lower than in Ctrl. At the same time, no differences were found between Ctrl and the groups receiving AM251 alone or in combination with AEA. In addition, a deeper investigation obtained using the Tg*(mpeg1:EGFP)*^*gl22*^ transgenic xenografts provided evidence that the reduction of vascularization is not caused by different recruitment of macrophages around the tumor area since a similar number was found in all experimental groups (Supplementary Fig. 2).

### Evaluation of HCT116 cell metastatic potential in xenografts

**Fig. 3:**
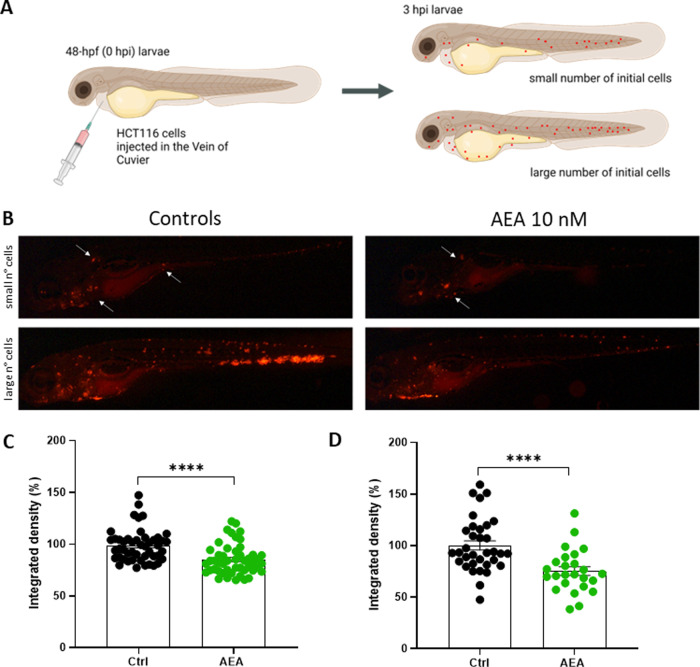
AEA exposure reduces HCT116 metastatic ability. **A** HCT116 cells were directly injected into the Vein of Cuvier of 48-hpf WT larvae. The image shows the experimental procedure and the division of xenografts considering the number of HCT116 circulating cells 3 hpi. Image created with Biorender.com. **B** Stereomicroscope Representative Images of 3dpi Ctrl- (on the left) and AEA exposed xenografts (on the right). White arrows pinpoint HCT116 cells in metastasis. **C**, **D** Integrated density of HCT116 metastatic tumor cells in 3 dpi zebrafish larvae injected with small (**C**) and large (**D**) numbers of cells. The mean value of the integrated density in Ctrl was set as 100%. The error bar indicates SEM. (Small: Ctrl *n* = 48; AEA *n* = 50. Large: Ctrl *n* = 34; AEA *n* = 25 from three independent experiments). Statistical analysis was performed using Student’s *t*-test. ****p < 0.0001.

These results set the basis for a more detailed analysis of the effect of AEA on the occurrence of metastasis. Therefore HCT116 tumor cells were directly injected into the Cuvier’s vein, thus forcing the metastasis appearance. Since evidence regarding the specificity of the AEA effect was gained by performing experiments with its inhibitor AM251, for this experiment only two experimental groups were considered: Ctrl and AEA. Moreover, since the precise control of the number of HCT116 cells injected in the Cuvier’s vein results sometimes very challenging, 3 h after the injection (3 hpi), the animals were divided into two sub-groups on the basis of the number of circulating cells injected: small and large (Fig. 3A). Xenograft from each group was randomly divided between the two experimental conditions, Ctrl and AEA treated. At 3 dpi, the number of metastatic cells present in the animal (as the quantity of red fluorescence detectable). As shown in Fig. 3C and D, AEA treatment reduces metastasis growth when the tumor cells are directly placed into blood circulation.

### Zebrafish and HCT116 RNA-seq and RT-PCR Validation

Starting from the results above described, obtained by confocal microscopy investigation, RNA sequencing analysis was performed to unveil molecular markers involved in tumor cell proliferation, angiogenesis, and immune response. Since a strong variability among replicates was observed, one replicate per experimental group was discarded. The choice of the samples to discard was based on the overall clustering of all the samples after principal component analysis and not only on the clustering in specific comparisons.

**Fig. 4:**
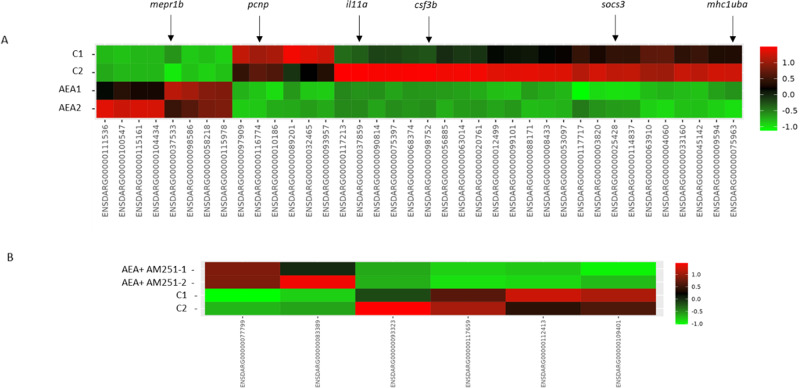
Heatmaps showing the list of DEG in zebrafish xenograft exposed to AEA and AEA + AM251. **A** AEA vs. Ctrl. **B** AEA + AM251 vs. Ctrl. Color scales represent fold-change between two groups as described (green = downregulation, red = upregulation). Only significant DEGs (FDR < = 0.05) have been included. Arrows show DEGs analyzed in this study.

Considering the *Danio rerio* genome, with respect to Ctrl, 38 differentially expressed genes (DEGs) were found in AEA larvae, 8 upregulated and 30 downregulated and 6 DEGs with respect to AEA + AM251 larvae, 2 upregulated and 4 downregulated. No DEGs were found between AM251 and Ctrl larvae (see Heatmaps, Fig. 4).

Regarding the *Homo sapiens* genome, no DEGs were found among different experimental groups. RNAseq data can be accessed through NCBI Bioproject—ID PRJNA911178

Nevertheless, the accuracy and reliability of RNA seq results were supported by a series of Real-Time PCRs on either DEGs, false-discovery rate (FDR) < 0.05, or not statistically significant genes (FDR ≥ 0.05) selected considering their role in cell proliferation, angiogenesis, and immune system (*vegfaa, vegfab, vegfd, mep1b, socs3, csf3b, il11a, mhcIuba, pcnp, casp3*). Details regarding the description of the selected genes are reported in Supplementary Table 1.

PCR results among different experimental groups are reported in Supplementary materials. As shown in Supplementary Figs. 3 and 4, the relative abundance in a reverse transcription-quantitative polymerase chain reaction (RT-qPCR) was consistent with RNA-Seq results, suggesting that the transcript identification and quantification were highly consistent between the two techniques. Genes selected from RNASeq analysis were in good accordance with the expression intensities by RT-qPCR.

### Molecular analysis of biomarkers involved in tumor cell survival/growth

**Fig. 5:**
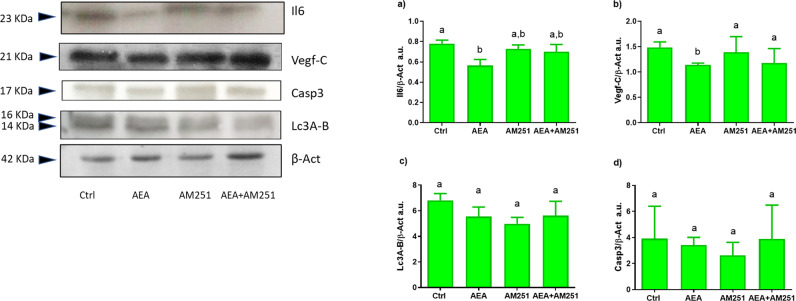
AEA effects on molecular targets involved in angiogenesis and inflammation. Insets show a representative Il6, Vegf-C, Casp3, Lc3A/B, and β-Act Western Blot in the different experimental groups. Densitometric analysis of three independent experiments, a.u. (arbitrary units) **a** Il6, **b** Vegf-C, **c** Lc3A/B, **d** Casp3. Different letters above each column indicate statistical differences among groups (one-way ANOVA, *P* < 0.05).

Western blot analysis on WT xenografts of Lc3A/B and Casp3 did not show any significant changes among experimental groups. On the contrary, Vegf-C and Il6 protein analysis revealed a significant reduction in xenograft exposed to AEA. No significant changes were induced in xenografts exposed to AM251 alone or in combination with AEA (Fig. 5).

### In vitro AEA effect on HCT116 cell proliferation

To investigate the effects of AEA exposure on cell viability and proliferation, HCT116 cells were reared with five different AEA concentrations in the range from 0.005 to 5 μM. In vitro tests were carried out in low serum concentrations (i.e., 0.5% serum) to limit cellular growth and increase cell responsiveness to the compound. Indeed, the effect of cannabinoids is cell context-dependent, being modulated by the presence of growth factors [17]. Cell viability was evaluated using the CCK-8 assay after 72 h of treatment but in contrast to what was previously observed in animal models, all AEA concentrations had no anti-proliferative activity (Supplementary Fig. 5).

### Multivariate statistical analysis

**Fig. 6:**
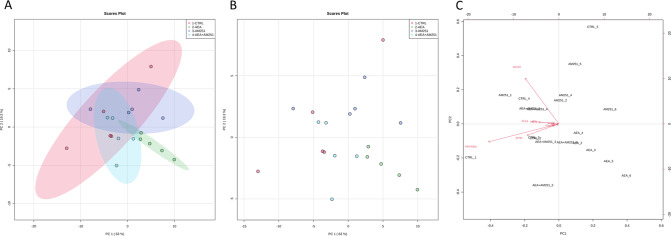
PCA showing the clear segregation of AEA xenograft and the overlapping of Ctrl, AM251 e AEA + AM251 groups. Score plot of Ctrl (red), AEA (green), AM251 (blue), and AEA + AM251 (light blue) **A** with 95% confidence region or **B** without 95% confidence region. Axes show scores on PC1 (63%) and PC2 (19.3%). **C** Biplot shows the loading analysis with a red arrow representing the variables. The bottom and left axes show scores on PC1 and PC2, top and right axes show loading values.

PCA analysis was computed to visualize the entire dataset of gene expression. This technique allows the visualization of samples within groups as single points, plotted in a two-dimensional space built with the calculated PCs. PC scores are used to determine the position of the samples in the 2D space. PC’s order indicates their importance in the dataset, with PC1 accounting for the highest amount of variation. From the PCA analysis emerged that Ctrl overlapped with AM251 and AEA + AM251 groups, while the AEA group clusters alone (Fig. 6A), with an overall satisfactory cumulative explained variance (68.8%), showing the ability of AM251 to restore the Ctrl condition when co-administered with AEA. A biplot was generated, showing the loading analysis together with the PCA (Fig. 6B). This analysis allows an understanding of the contribution of variables in building the model. The loading of each variable is displayed as red arrows and the name of each variable is shown (Fig. 6C). The length of the arrows is directly proportional to the contribution in the model and the distance of the arrow point from the central axes reflects their contribution to the PC1 or PC2. The two genes providing the most variability for the model are *socs3* and *mhcluba*, the first possesses a contribution at almost the same level to both PCs, and the second contributes more to PC1 than PC2. *csf3b* and *il11a*, contribute to almost the same extent to the PC1 with a very small contribution to PC2, while *pcnp* contribute to PC1 is appreciable from the plot with little contribution to PC2. Furthermore, *vegfc* and *mep1b* show only a slight effect only on PC1, together with a very little contribution of IL-6 to the model built inside the PC1.

## Discussion

Starting from the macroscopic evidence that xenografts exposed to AEA presented a reduced sub-intestinal vascularization, thus affecting tumor growth, the integration of results obtained by this multidisciplinary approach strongly suggested AEA ability to alter the tumor environment, causing less favorable conditions for the proliferation of tumor cells, which viability resulted not directly affected. In these xenografts, indeed, RNAseq revealed no differences among HCT116 cell transcripts regardless of the treatment and this evidence was further observed by in vitro cancer cell exposure to AEA. AEA exposure, indeed, did not affect cell viability at each of the concentrations tested and deeper analysis, focusing on the 10 nM concentration, revealed that the exposure does not affect cell proliferation and apoptosis (data not shown).

RNAseq analysis allowed the identification of a set of transcripts that cooperate to create the proper microenvironment suited for HCT116 cell proliferation and tumor vascularization. Among DEGs, the suppressor of cytokine signaling 3, s*ocs3*, was selected considering the cancerogenic role of the Socs3 protein in the development of colorectal-, breast-, and ovarian cancer [18–20]. Several studies described its role in the regulation of key proteins involved in inflammation, angiogenesis, and cell survival, including Vegf, Il-6, and Bcl-2 [21–23], by activating the Il-6/Jak/Stat3 pathway [24]. Thus, the results obtained in this study suggested that AEA by downregulating zebrafish *socs3* mRNA possibly contributes to the decrease of Il6 and Vegf proteins found in xenografts exposed to AEA, measured by Western blot analysis, probably affecting the Il6/Jak/Stat3 signaling. The key role of this signal in the observed results was also confirmed by PCA. Nevertheless, RNA-seq showed a decreasing trend, although not significant, of *jak1* and *stat3* mRNA, resulting in line with studies reporting the antiproliferative effect of AEA [9, 25, 26]. Concerning Vegf family members, studies reported that a lower expression of *Vegf* factor concurs with the reduction of tumor volume affecting angiogenesis [27–29]. Therefore, as previously observed in vitro [30] and in a mouse xenograft model [30], it can be assumed that AEA might regulate tumor proliferation by reducing VEGF levels, thus affecting the VEGF/VEGFR2- binding between endothelial- and HCT116 cells. Moreover, VEGF can also be produced by tumor-associated macrophages (TAMs) which under hypoxic conditions [31], secrete VEGF [32]. In this regard, although no changes in macrophage number were observed, it could be supposed that AEA may not affect TAM migration, thus increasing their number, but only VEGF secretion [33, 34] contributing to the creation of a less favorable environment for tumor cell growth. Indeed it is known that HCT116 cells express high VEGFR levels [30], which guarantee their growth through paracrine/autocrine vascular endothelial signaling [35]. Indeed, in colorectal cancer, the high expression of *Vegf* is correlated with poor prognosis [36], and the downregulation of VEGF-C leads to the reduction of tumor-initiating cells and inhibition of metastasis [37], in this way supporting the lack of metastasis observed in our xenograft.

Among AEA-induced DEGs, also the colony-stimulating factor 3 (granulocyte) b *(csf3b)*, a pleiotropic cytokine that promotes the activation of monocytes and macrophages [38], contributes to angiogenesis by synthesizing VEGF, specifically at the early stage of primitive endothelial tubule formation [39]. Thus, its downregulation could contribute to the observed Vegf-C protein decrease.

Several studies reported that AEA treatments could reduce inflammation via the regulation of inflammatory target genes and activation of inflammasome components [40, 41]. In this contest, both RNA-seq analysis and Western blot data reported a decrease in Il6 levels, supporting AEA anti-inflammatory activity. Il6 high production, indeed, causes a rich inflammatory environment and can promote the malignant transformation of cancer cells supporting their proliferation, survival, and metastatic dissemination [42–44]. It is known that IL6 leads to STAT3 phosphorylation and dimerization by acting on transcription of antiapoptotic-, angiogenic-, proliferating-, and immune response factors including BCL-2, BCL-xL, VEGF, and MMP2/9 [45].

In this regard, in this study, both human and zebrafish transcriptome showed a *bcl-2* downregulation, although not statistically significant, which could be associated with Il6 reduction, resulting in agreement with a previous study in *IL6*^*−/−*^ mouse [46]. The lack of significant changes in *bcl2* levels herein observed suggests a marginal role of apoptosis in either tumor physiology or larval growth. This result is endorsed by Caspase3 analysis, in which mRNA and protein levels, did not change among experimental groups, suggesting that apoptosis has a marginal role in this specific phase of xenograft physiology.

Recently, a novel nuclear factor involved in cell cycle regulation, transcription, and apoptosis was identified, the PEST-containing nuclear protein (PCNP). This factor degrades residual proteins linked with tumor cell growth and differentiation in a tissue-specific manner [47, 48]. In this light, the downregulation of zebrafish *pcnp* we measured might be associated with the decrease, although not significant, of *stat3* and *stat5*, affecting the Il6/Jak/Stat3 signaling, above described.

In addition to Il6, AEA treatment affects also *il11* transcript level. Il11 is a cytokine related to the Il6 cytokine family controlling the release of inhibitory nuclear factor NF-kB which serves as a transcriptional activator of proinflammatory cytokines. It has been detected in many organs including gastrointestinal tracts and the liver [49, 50] and it has been shown to promote the progression of CRC [51] and prostate cancer [52]. For the first time, in our model, it was shown that AEA administration induces a significant downregulation of *il11*, possibly affecting the Il11/Stat3 pathway. This result agrees with previous data describing the inhibition of JAKs and STAT family members and interleukins by endocannabinoids and suggesting a role as a molecular entity with strong anti-tumorigenesis potentiality [51].

An additional process analyzed was autophagy, which is considered a double-edged sword since it can regulate tumor suppression and tumor cell survival [53].

In this study, LC3 protein levels do not change significantly among experimental groups. However, two possible scenarios regulated by autophagy can be assumed in our xenografts: in control and groups exposed to AM251, autophagy regulates metabolite uptake favoring HCT116 proliferation, as previously observed in different cancer cell lines [54, 55] while in AEA-exposed xenografts, it can be assumed that autophagy exerts a cytoprotective, tissue-protective and anti-inflammatory action [56, 57], as supported by the reduction of proinflammatory chemokines and in agreement with the results with human keratinocytes exposed to AEA [58]. Moreover, the Major histocompatibility complex (MHC) is one of the protagonists in the immune response and the recognition of non-self expressed on the membrane of all nucleated cells. The principal function of MHC-I is the presentation of peptide antigens to CD8^+^ T cells triggering their differentiation [59] and thus aiding the immune response. RNA-seq analysis on zebrafish transcriptome revealed the downregulation of *mhcIuba* transcript, which shares a high synteny with mammalian MHC [60] and suggests that AEA treatment activates the Natural Killer mediated immune response, thus promoting tumor cell death [61]. However, several studies are still required since the control of AEA on recognition of non-self by MHC-I should be deepened. Based on our transcript results, we can speculate that its reduction could be correlated to the decrease of *il11* mRNA levels, as recently demonstrated in the HD11 cell line [62]. It could be interesting to investigate how endocannabinoids can mitigate the antigen-immune response, not only focusing on the control of inflammation, but also on the regulation between self and non-self.

Our results evidenced changes in the expression of genes belong the metalloprotease family. The metalloproteases (ADAMs) are extracellular proteases involved in connective tissue homeostasis, intestinal barrier function, and immunological processes. Among them, meprins have a particular structure and functional features: meprinα is a pro-angiogenic enzyme and promotes tumor progression, while meprin is mainly related to some diseases [63]. The substrates for the metalloproteases are many transmembrane proteins such as pro-inflammatory cytokines including TNF-a and IL6R [64, 65]. Moreover, meprins balance the immune environment modulating the activity of IL1B, IL18, and IL6 which are the primary products released in response to tissue injury and inflammation [66, 67]. They are also involved in ECM remodeling when an imbalance of cytokines and mononuclear cells occurs in response to mechanical stress or ROS production [68]. Surprisingly, in this study, the RNA-seq analysis revealed an increase of *mep1b* gene expression, which, as reported in several studies, should be associated with an increase of cytokines by the leukocyte influx promotion [67] and tumor cell migration [69]. Conversely, among DEG, Ils resulted downregulated, suggesting that in our xenograft model, *mep1b* could be only involved in larval development, possibly controlling the active cellular progression typical of an early stage larva.

Collectively, our data suggest a pivotal role of AEA in the anti-angiogenic, anti-proliferative, and anti-inflammatory process in intercellular tumor-endothelial cell communication resulting in the containment of tumor and evidenced that zebrafish larvae xenografts constitute a promising fast assay for precision medicine, bridging the gap between genotype and phenotype in an in vivo setting.

## Materials/subjects and methods

### Experimental model

Wild-type Tuebingen (WT), Tg(*fli1*:EGFP)^y1^ [70], and Tg(*mpeg1:*EGFP)^gl22^ [71] transgenic zebrafish lines were used for the different analyses. Tg(fli1:EGFP) ^y1^ [70] expresses the EGFP under the control of the promotor of the *fli1* gene, an endothelial cell marker [72] while the Tg(*mpeg1*:EGFP)^gl22^ line presents a Green Fluorescent macrophage population. Fish were maintained according to standard procedure. Embryos were obtained from natural spawning and raised at 28.5 °C in a 12:12 light:dark (LD) cycle in fish water (5 mM NaCl; 0.17 mM KCl; 0.33 CaCl_2_; 0.3 mM MgSO_4_; pH = 7). All husbandry and experimental procedures complied with the Italian and European Legislation for the Protection of Animals used for Scientific Purposes (Directive 2010/63/EU). The age of embryos is indicated as hours post-fertilization (hpf), hours post-injection (hpi), and days post-fertilization (dpf) for all experimental data shown.

### Cell culture and labeling

HCT116 cells were selected considering their dedifferentiated phenotype, invasive and metastatic potential, and clinical significance [73]. Cells were tested for mycoplasma (VenorGeM Classic, Minerva Biolabs GmbH, # 11-1025) and cultured in RPMI Medium 1640 with GlutaMAX-I (Thermo Fisher Scientific, Waltham, USA, # 61870036,) supplemented with 10% fetal bovine serum (FBS) (Thermo Fisher Scientific, Waltham, USA, #10270106) in a humidified atmosphere containing 5% of CO_2_ at 37 °C. For xenotransplantation, cells were washed, trypsinized, and re-suspended in RPMI medium containing Dil Vybrant Red Fluorescent dye (Thermo Fisher Scientific, Waltham, USA, #V22885) for 20 min at 37 °C. Cells were then centrifuged at 1200 rpm for 5 min, the supernatant was discarded, and cells were washed two times with PBS. Finally, cells were re-suspended in PBS for injection in the zebrafish yolk.

### Xenotransplantation

Wild type (WT), Tg(*fli1*:EGFP)^y1^, and Tg(*mpeg1*:EGFP)^gl22^ zebrafish embryos at 48 hpf were immobilized with 0.04% tricaine (Merck KGaA, Darmstad, Germany, #E10521) and positioned on a thick film of 2% agarose in fish water. To investigate the tumor volume and interaction with the host and the therapeutic molecules, approximately 200–400 Dil-labeled (VybrantTM DiI) HCT116 cells were injected into the inferior section of the yolk sac (Supplementary Fig. 1). To investigate the metastatic potential of the tumor, the same number of cells were injected directly into the Cuvier’s vein. After injection, xenografts were maintained at 35 °C until the end of the experiments. At 3 hpi, unsuccessfully injected xenografts were discarded [74].

### In vivo and in vitro drug administration

Arachidonylethanolamide (Anandamide—AEA) (Merck KGaA, Darmstad, Germany, #94421-68-8) and 1-(2,4-Dichlorophenyl)-5-(4-iodophenyl)-4-methyl-N-1-piperidinyl-1H-pyrazole-3-carboxamide (AM251) (Merck KGaA, Darmstad, Germany, # 183232-66-8), were dissolved in Ethanol 100% and further diluted in fish water and used at 10 nM as previously described in [75, 76].

### In vivo exposure

At 6 hpi zebrafish xenografts were randomly divided into four experimental groups:

- Controls (Ctrl): exposed to Ethanol (EtOH)
- AEA: exposed to 10 nM AEA
- AM251: exposed to 10 nM AM251
- AEA + AM251: exposed 10 nM AEA + 10 nM AM251

Chemicals were daily replaced till 4 dpf. The control group received the same amount of EtOH used to dissolve chemicals.

The sample size for in vivo experiments is shown in figure legends throughout the manuscript. The size of the samples used in zebrafish experiments was determined by the alpha error of 0.05 and the statistical power of 95%. The minimum number of 6 controls and 6 mutated/treated samples was implied by the sample population’s required size (Sample Size Calculator, ClinCalc.com). The number of individual embryos that could be made available for each experiment after fluorescence screening also had an impact on sample size.

Gross congenital abnormalities, which typically affect 5–10% of an egg clutch, were excluded from the analysis. The purpose of the data exclusion is only to collect biologically meaningful phenotypes resulting from the specific treatment and not from sporadic changes that are frequently seen in wild animals.

### In vitro exposure

Cell viability was determined using the cell counting Kit-8 colorimetric assay (CCK-8, Bimake, USA, # B34304). HCT 116 cells were seeded in 96-well plates with RPMI 1640 (Thermo Fisher Scientific, Waltham, USA, # 61870010) and 10% fetal bovine serum (FBS) at a density of 5 × 10^3^ cells/well. After 24 h of incubation at 37 °C, the medium was removed and a new culture medium containing 0.5% FBS and AEA were added. AEA, in EtOH, was directly dissolved in cell culture medium to 50 µM solution and then serially diluted to give a final test concentration of 5, 10, 100 nM, and 1 and 5 µM. Concentrations were selected based on previous in vitro studies evaluating the anti-carcinogenic AEA properties in different cell lines [77, 78]. Cells were treated at 37 °C for 72 h changing the medium every day, after which cell viability was measured. Briefly, 10 µl of CCK-8 reagent was added to each well and incubated for 30 min at 37 °C, then absorbance at 450 nm was measured using a microplate reader (Infinite F200 PRO, Tecan, Männedorf, Switzerland). The absorbance in the control group was regarded as 100% cell viability. The percentage of viability was calculated using the formula: “[OD (optical density) of treated cells background absorbance/OD of untreated cells (control)—background absorbance] × 100”. All controls and samples were measured by four independent experiments with five replicates for each concentration. Values are expressed as the mean ± SD.

### Live imaging

Totally, 3 dpi WT, Tg(*fli1*:EGFP)^y1^ and Tg(*mpeg1*:EGFP)^gl22^ xenografts were anesthetized with 0.04% tricaine, embedded in 1% low-melting agarose, placed on a depression slide and analyzed by confocal microscopy. The Nikon C2 confocal system (Nikon, Minato, Japan), along with the software NIS ELEMENTS, was used to record images. At 3 dpi, WT xenografts injected in the Cuvier’s vein were anesthetized with 0.04% tricaine, embedded in 1% low-melting agarose, placed on a depression slide and analyzed with Leica DMR fluorescent microscope (Leica, Wetzlar, Germany) and the Nikon DS-Fi2 digital camera.

### Tumor volume, angiogenesis, and metastatic potential quantification

All images of WT, Tg(*fli1*:EGFP)^y1^, and Tg(*mpeg1*:EGFP)^gl22^ xenografts obtained with the Nikon C2 confocal system were analyzed using Fiji imaging software (https://imagej.net/software/fiji/). The tumor size was obtained by calculating each Z stack’s tumor area (red fluorescent structure), while the angiogenesis quantification was performed using the image calculator process. To identify only endothelial cells, HCT116 fluorescence signals were subtracted from *fli1*-eGFP signals. Total signal intensity was calculated according to previous work [79]. Macrophage analyses using the Tg(*mpeg1*:EGFP)^gl22^ xenografts were performed by counting the number of macrophages present in the area surrounding the tumor. An equal area was selected for all the samples. The percentage of animals with micrometastases was calculated by counting the number of 3-dpi larvae with micrometastases in all the different treatment conditions, despite being injected exclusively in the yolk. The total fluorescence of the metastasis of animals injected directly in the Cuvier’s vein was analyzed with the imaging software Fiji, to observe the effect of therapeutic molecules on the proliferation of metastases.

### RNA extraction, cDNA synthesis, and Real-time PCR

Total RNA was extracted from 5 pools of ±20 larvae for each experimental group, using RNAzol RT (Merck KGaA, Darmstad, Germany, # R4533). Genomic DNA was removed by DNase I digestion (Merck KGaA, Darmstad, Germany, # AMPD1). RNA concentrations were determined by nanophotometer P330 (Implen, München, Germany) and integrity was assayed with Bioanalyzer (Agilent, CA, USA). Part of the total RNA was used for cDNA synthesis, and part was to create a library for RNA-seq. The reverse transcription was conducted from 1 μg total RNA with High-Capacity cDNA Reverse Transcription Kit (Applied Biosystems, Waltham, Massachusetts, USA). The qRT-PCRs were performed with the SYBR green method in a CFX thermal cycler (Bio-rad, Milan, Italy) as previously described [80]. For each experimental group, replicates (N = 5) were run in duplicate. The final primer concentration was 10 pmol/μL. Ribosomal protein 13 (*rpl13*) and ribosomal protein 0 (*rplp0*) mRNAs were used concomitantly to normalize target genes expression levels analyzed by CFX Manager Software version 3.1 (Bio-Rad), including GeneEx Macro Conversion and GenEx Macro files and results are represented by bar-plots along with the standard deviation. Specific primer pairs for target genes (Supplementary Table 1) were designed with Primer-Blast.

### RNA-seq analysis and differential expression analysis of *Homo sapiens*/*D. rerio* xenografts

The starting dataset included RNA-seq reads from 12 samples belonging to the four experimental groups, i.e., Ctrl, AEA, AM251, and AEA + AM251. The quality of the reads was assessed with the software FASTQC (https://www.bioinformatics.babraham.ac.uk/projects/fastqc/) then a trimming step was performed in order to remove adapters and low-quality bases from the reads. The following parameters were used: the minimum length was set to 35 bp and the quality score to 25. The software TRIMMOMATIC [81] was used for this scope. On average, 140 million filtered reads were obtained per sample.

The high-quality reads were aligned against the *Homo sapiens* genome (GRCh38–104) and *D. rerio* genome (GRCz11–105) with STAR aligner (version 2.7.9a) [82]. On average, 0.74% of the total reads could be mapped uniquely on the human genome and 81.76% of the reads could be mapped uniquely on the zebrafish genome. In order to perform disambiguation between the two species, the software disambiguate was used. FeatureCounts (version 2.0.0) [83] was used to calculate gene expression values as raw fragment counts. In addition, a normalization was applied to the raw fragment counts by using the trimmed mean of *M*-values and the fragments per kilobase million normalizations.

### Western blot analysis

Whole larval homogenates from 3 different pools of 15–20 larvae per group, were electrophoresed and transferred to PVDF as previously described [84]. Briefly, 7 mg of each protein sample was separated using 4% stacking and 10% separating sodium dodecyl sulfate-polyacrylamide gel electrophoresis and electroblotted onto a filter using a mini trans-blot electrophoretic transfer cell (Bio-Rad, Milan, Italy). The transfer was carried out for 30 min using Trans-Blot TurboTM Transfer System. After blocking in 2% bovine serum albumin (BSA; Merck KGaA, Darmstad, Germany, # A2153) in PBS. The following primary antibody was used: LC3A/B (Cell Signaling, Beverly, MA, USA, #BK4108S), Caspase 3 (Cell Signaling, Beverly, MA, USA, # BK9661S), IL6 (Abcam, Cambridge, UK, #ab208113,) and VEGF-C (Cell Signaling, Beverly, MA, USA, # BK2445S,) antibodies were diluted 1:1000 in a solution containing 2% BSA and 0.1% TWEEN 20 in PBS and incubated overnight at 4 °C. β-actin antibody (Cell Signaling, Beverly, MA, USA, # BK4967S) was used as internal standard. The reaction was visualized with ECL-PLUS (GE Healthcare, Milano, Italy) chemiluminescent reagent for Western blotting. Densitometric analysis was performed using Fiji software for Windows.

### Statistical analysis

RNA-seq statistical analyses were performed with R with the package edgeR. The edgeR package determines differential expression using empirical Bayes estimation and exact tests based on a negative binomial model. Genes with no expression, i.e., zero counts, across all the samples were discarded. The genes showing an FDR lower or equal to 0.05 were considered statistically significant.

qPCR, Western blot, and imaging statistical analysis were performed with Graph Pad Prism V9.0.1. (GraphPad Software, Inc., San Diego, CA, USA). Data of all groups were normally distributed as assessed using Shapiro–Wilk’s test (*p* > 0.05), and there was homogeneity of variances, as assessed using Levene’s test for equality of variances (*p* > 0.05). Data are presented as means ± SEM or ±SD and were analyzed by one-way ANOVA followed by Dunnett’s multiple comparison test. When the collected data was expressed in percentage, arcsin transformation was conducted before ANOVA. Asterisks (*) or different letters on histogram bars indicate statistically significant changes among groups. The *p*-values were set as *p* < 0.05.

Metaboanalyst 5.0 online platform (University of Aberta, Edmonton, AB, Canada) was used to perform Unsupervised PCA on a set of genes with *p*-value < 0.05 after normalization by a median, log transformation, and Pareto scaling of the data set. To visualize the separation among groups, a 2D score plot was generated plotting the first two principal components and a biplot was computed to obtain information on the importance of the variables in the projection.

## Supplementary information

Reproducibility checklist

Supplementary Info

Original Western blot

## Data Availability

All data are presented in the main manuscript or the supplementary file. Additional information will be provided by the corresponding author upon reasonable request.
