# Supplementary material for: A zebrafish HCT116 xenograft model to predict anandamide outcomes on colorectal cancer
Source: Cell Death Dis. 2022 Dec 23;13(12):1069. doi: 10.1038/s41419-022-05523-z (PMC9789132; doi:10.1038/s41419-022-05523-z)
Supplement: Supplementary file 2 — Supplementary Info [file 41419_2022_5523_MOESM2_ESM.docx]

**Supplementary Information to Maradonna et al.,**

**Optimization of the optimal cell number for transplantation**

Preliminary experiments were performed to identify the optimal number of HCT116 cells to be used in the transplantation experiment. Following previous studies, embryos were injected with 200-400 or 500-1000 cells (1–3).

Results showed that only in fish transplanted with 200-400 cell, AEA concentration significantly reduced tumor cell proliferation, thus experiments were performed using this cell number.





**Supplementary Figure 1**. **HCT116 tumor size (3 dpi) in WT xenografts injected with 200-400 cells and 500-1000 cells measured as total Dil Vibrant Red fluorescence.** Each trial consisted of a Ctrl and a AEA exposed group. The mean value of the tumor size in the Ctrl was settled as 100%. Error bar indicates SEM (200-400 cells, Ctrl N = 17 AEA N = 12; 500-1000 cells, Ctrl N= 11 AEA N=15). Statistical analysis was performed using T-test analysis . *, P < 0.05.

**Macrophage quantification**





**Supplementary Figure 2. Macrophage analysis in *mpeg1*:EGFP larvae (3 dpi) injected with HCT116 cells**. Quantification of the number of macrophages (GFP-positive cells) in a similar region of interest (ROI) size. The fluorescence of each individual was normalized for their tumour volume. The mean number of macrophages in Ctrl was set as 100%. Error bar indicates SEM (Ctrl N = 10; AEA N=9; AM251 N=8; AEA + AM251 N=11). Sample size derived from three independent experiments.

**RNA-seq validation by RT-PCR**

DEGs of interest were quantified by Real Time PCR to validate RNA seq results. csf3b, il11, *socs3, mhcluba,* and *pcnp*  mRNA expression resulted significant downregulated in xenografts exposed to AEA. Both AM251 and AEA+AM251 groups presented mRNA levels similar to those measured in Ctrl fish. Among selected genes, only *mep1b* expression resulted significantly upregulated in AEA exposed fish (Supplementary Figure 3 a-f). Lack of significant change was observed concerning *casp3 (Supplementary Figure 4a), vegfaa, vegfab and vegfc* mRNA levels, according the RNA seq analysis. Nevertheless, although not statistically significant, vegfs mRNA resulted downregulated by AEA treatment (Supplementary Figure 4 b-d).





**Supplementary Figure 3**. **Real time PCR analysis on selected DEGs**. *Soc3* (**a**), *csf3b* (**b**), *mhcIuba* (**c**), *mep1b* (**d**), *pcnp* (**e**), *il11a* (**f**) mRNA levels xenograft, normalized against *rplp0* and *rpl13a*. Data are shown as mean ± SD (*N* = 5). Asterisk * above each column denote significant differences among the experimental groups (*P* < 0.05), which have been analyzed by one-way ANOVA followed by Tukey’s multiple comparison test.





**Supplementary Figure 4.** *casp3* (**a**), *vegfaa* (**b**), *vegfab* (**c**), *vegfd* (**d**) mRNA levels in xenograft, normalized against *rplp0* and *rpl13a.* Data are shown as mean ± SD (*N* = 5). Asterisk * above each column denote significant differences among the experimental groups (*P* < 0.05), which have been analyzed by one-way ANOVA followed by Tukey’s multiple comparison test.

*In vitro* viability of cell exposed to grade concentration of AEA


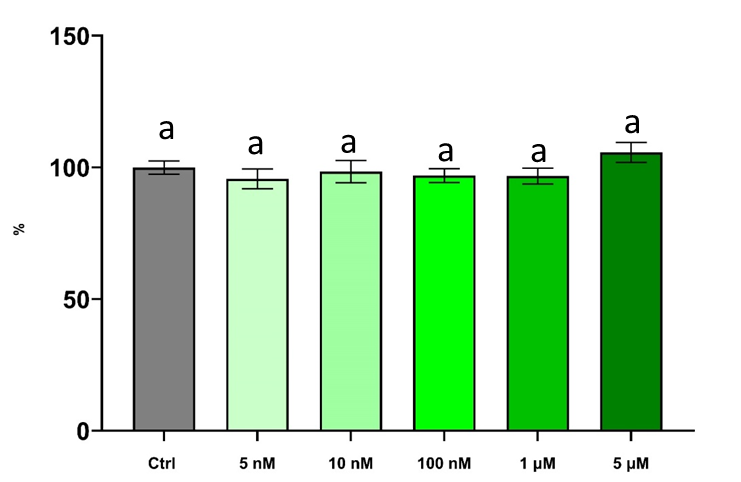


**Supplementary Figure 5.** **AEA does not affect HTC116 cell viability in vitro**. Bar plot showing cell proliferation in HCT116 cells exposed to different AEA concentration. The same letter (a) above each column denotes any significant difference among experimental groups (P>0.05)

| **Gene name** | **geneid** | **description** | **Kegg pathway** |
| --- | --- | --- | --- |
| *pcnp* | ENSDARG00000116774 | PEST proteolytic signal-containing nuclear protein-like [Source:NCBI gene;Acc:110437793] | NF-kappa B signaling pathway |
| *socs3a* | ENSDARG00000025428 | suppressor of cytokine signaling 3a [Source:ZFIN;Acc:ZDB-GENE-030131-7349] | TNF signaling pathway |
| *mep1b* | ENSDARG00000037533 | meprin A, beta [Source:ZFIN;Acc:ZDB-GENE-041014-209] | Protein digestion and absorption |
| *il11a* | ENSDARG00000037859 | interleukin 11a [Source:ZFIN;Acc:ZDB-GENE-051019-1] | Cytokine-cytokine receptor interaction |
| *mhc1uba* | ENSDARG00000075963 | major histocompatibility complex class I UBA [Source:ZFIN;Acc:ZDB-GENE-990415-145] | Natural killer cell mediated cytotoxicity |
| *csf3b* | ENSDARG00000098752 | colony stimulating factor 3 (granulocyte) b [Source:ZFIN;Acc:ZDB-GENE-141212-221] | IL-17 signaling pathway |
| *vegfaa* | ENSDARG00000103542 | vascular endothelial growth factor Aa [Source:NCBI gene;Acc:30682] | VEGF signaling pathway |
| *vegfab* | ENSDARG00000034700 | vascular endothelial growth factor Ab [Source:ZFIN;Acc:ZDB-GENE-030131-4605] | VEGF signaling pathway |
| *vegfd* | ENSDARG00000056624 | vascular endothelial growth factor D [Source:ZFIN;Acc:ZDB-GENE-070810-6] | VEGF signaling pathway |
| *casp3a* | ENSDARG00000017905 | caspase 3, apoptosis-related cysteine peptidase a [Source:ZFIN;Acc:ZDB-GENE-011210-1] | apoptosis |

**Supplementary Table 1**. Gene identification, description and related Kegg pathway

| **Gene Acronym** | **NCBI gene accession no.** | **Forward** | **Reverse** |
| --- | --- | --- | --- |
| ***rpl13a*** | NM_212784.1 | TCTGGAGGACTGTAAGAGGTATGC | AGACGCACAATCTTGAGAGCAG |
| ***rplp0*** | NM_131580.2 | CTGAACATCTCGCCCTTCTC | TAGCCGATCTGCAGACACAC |
| ***csf3b*** | NM_001143754.2 | ACACGCGGTGCAGTGTTTTT | GCCTGATTGTTCAACTGGGG |
| ***pcnp*** | NM_001326716.1 | TAGACTCAACAGCGTCTCTCTG | TGAGCGACTGATGCCTTGG |
| ***mhcIuba*** | NM_131471.1 | ACCTGGACTGCTGCTAATTCT | TAGCCCACATACTTCTGCACC |
| ***il11a*** | XM_693882.9 | GGGAGATGGTTCAGACCTCT | GATGTCCCGGTGCTTCCTA |
| ***mep1b*** | NM_001076621.2 | ACTTGATCTGGTGGAAGGAGACATT | AAGAAGTACGGCACAGTGGTAGG |
| ***socs3a*** | NM_199950.1 | GGAAGACAAGAGCCGAGACT | GCGATACACACCAAACCCTG |
| ***casp3a*** | NM_131877.3 | ACAGCAGTAACGCCACATTT | AGTTTGGGCTTTCCCACCAG |
| ***vegfaa*** | NM_001110349.2 | GACGTTTCGTGTCTCTGTCG | AAAAGAGTGCGTGCAAGACC |
| ***vegfab*** | NM_001328597.1 | GGACCTGCAGATGTGACAAA | ATCAAATCCTGTGCTCCGAG |
| ***vegfd*** | NM_001040178.1 | CTCAGTTCCTGCTCCCACTT | GGCCTCAACAGAGCTTCAAC |

**Supplementary Table 2.** Primer list

References

1. Cornet C, Dyballa S, Terriente J, Di Giacomo V. ZeOncoTest: Refining and automating the zebrafish xenograft model for drug discovery in cancer. Pharmaceuticals. 2020;13(1):1.

2. Ye S, Liu Y, Fuller AM, Katti R, Ciotti GE, Chor S, et al. TGFβ and hippo pathways cooperate to enhance sarcomagenesis and metastasis through the hyaluronan-mediated motility receptor (HMMR). Mol Cancer Res. 2020;18(4):560–73.

3. Fior R, Póvoa V, Mendes R V, Carvalho T, Gomes A, Figueiredo N, et al. Single-cell functional and chemosensitive profiling of combinatorial colorectal therapy in zebrafish xenografts. Proc Natl Acad Sci U S A. 2017;114(39):E8234–43.
