# Supplementary figures and images for: A zebrafish HCT116 xenograft model to predict anandamide outcomes on colorectal cancer
Source: Cell Death Dis. 2022 Dec 23;13(12):1069. doi: 10.1038/s41419-022-05523-z (PMC9789132; doi:10.1038/s41419-022-05523-z)

b-Actin


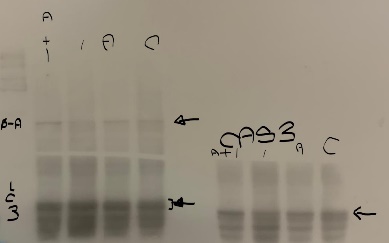

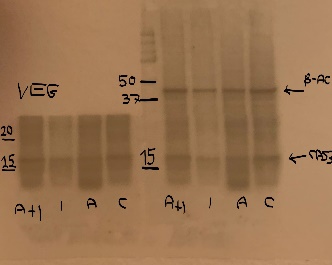

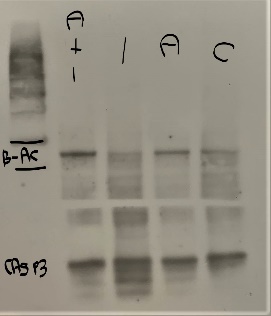

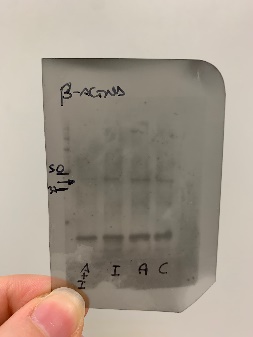


**Lc3**


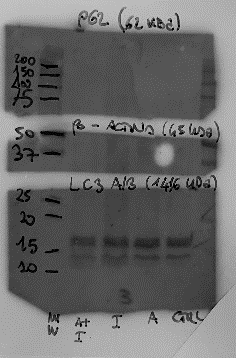

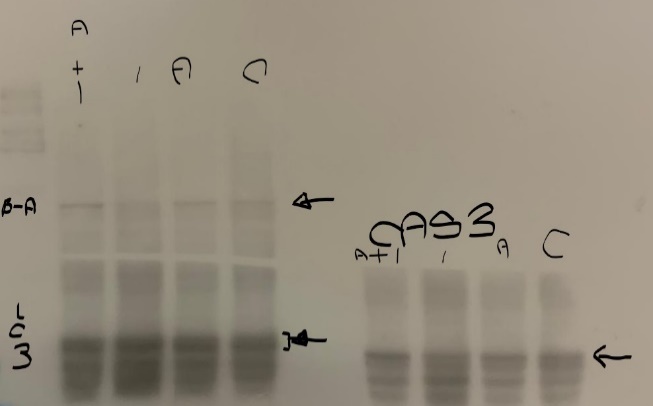


**Vegf-C**


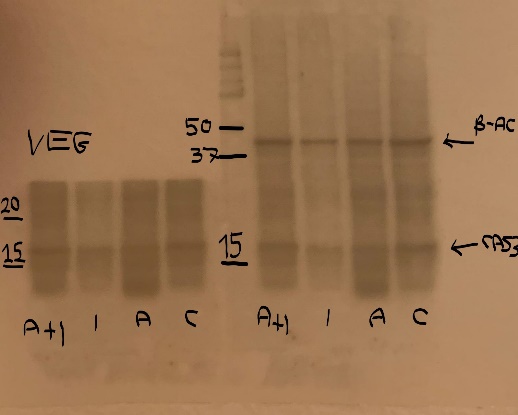

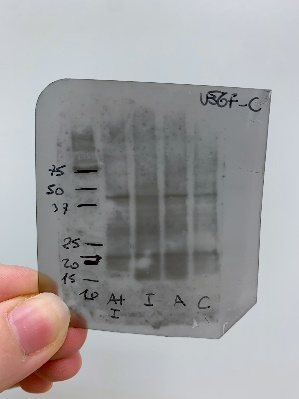

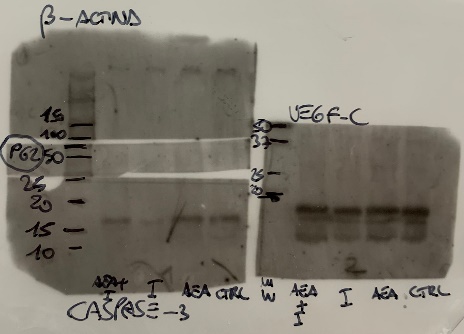


**
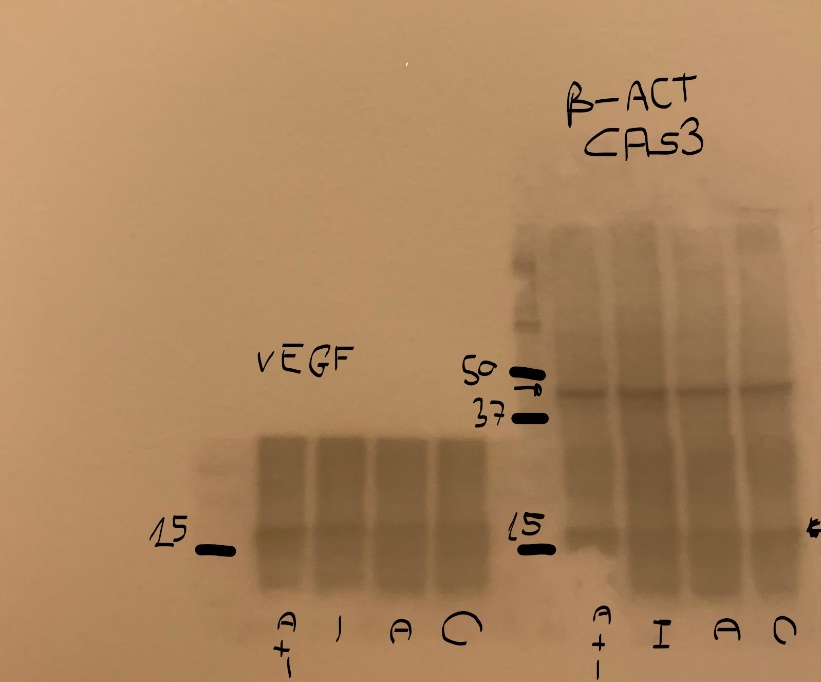
Casp3**


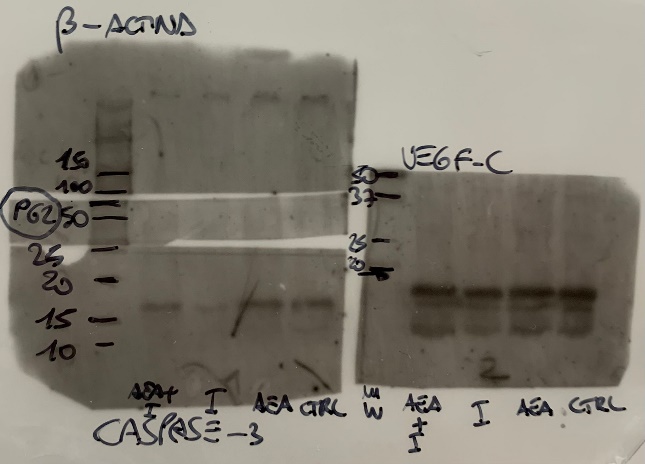

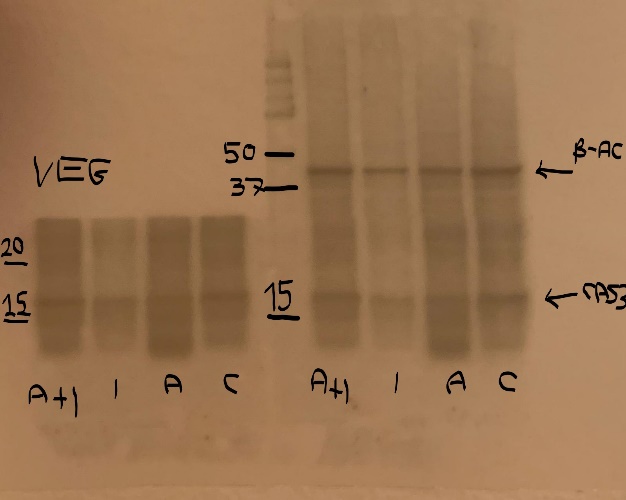

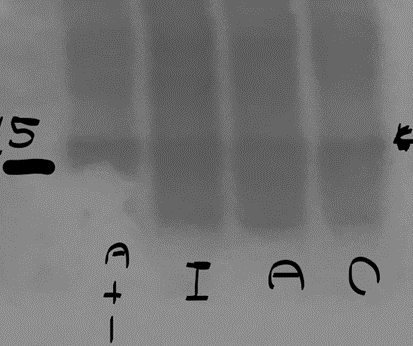


Il6


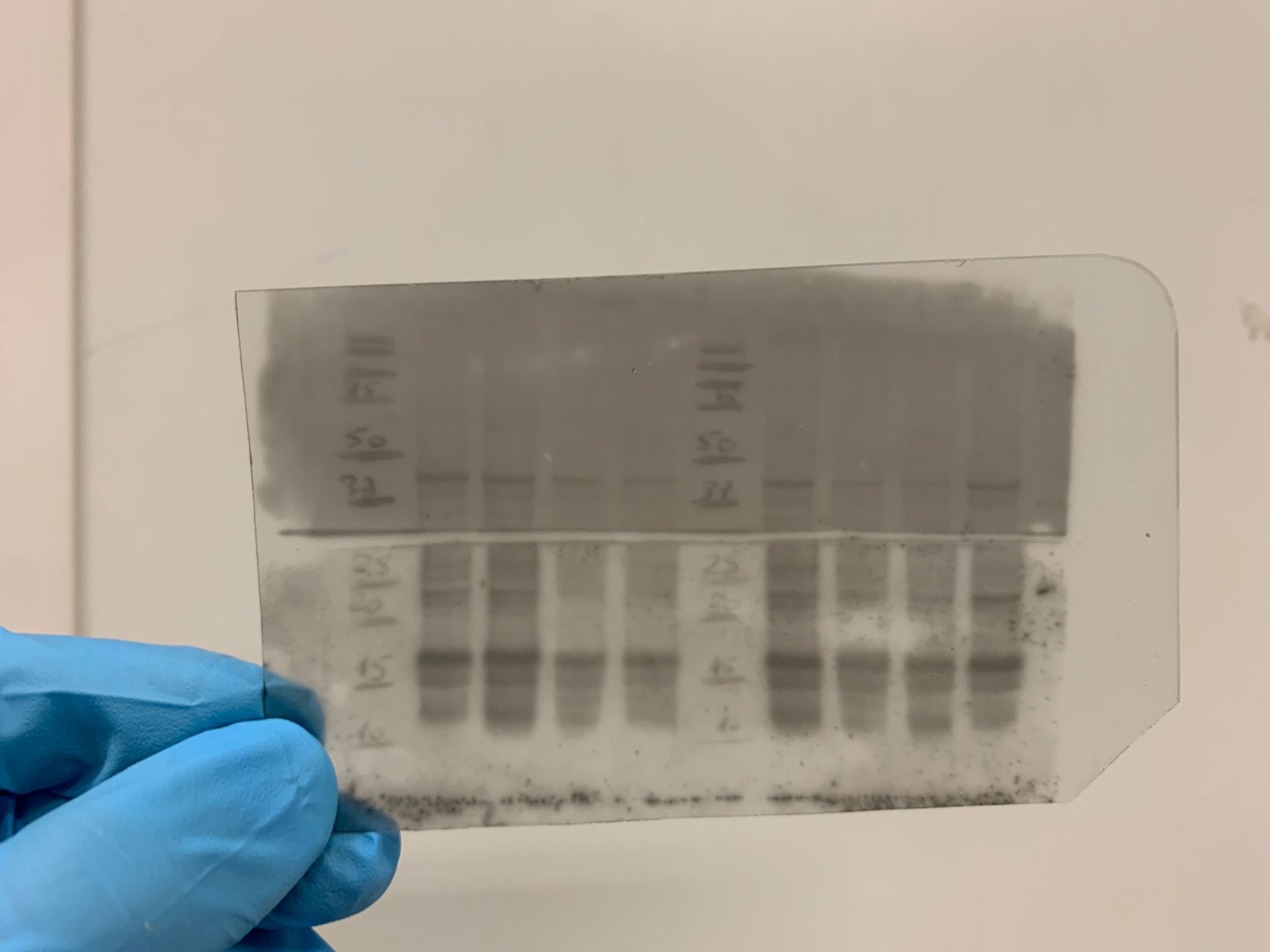


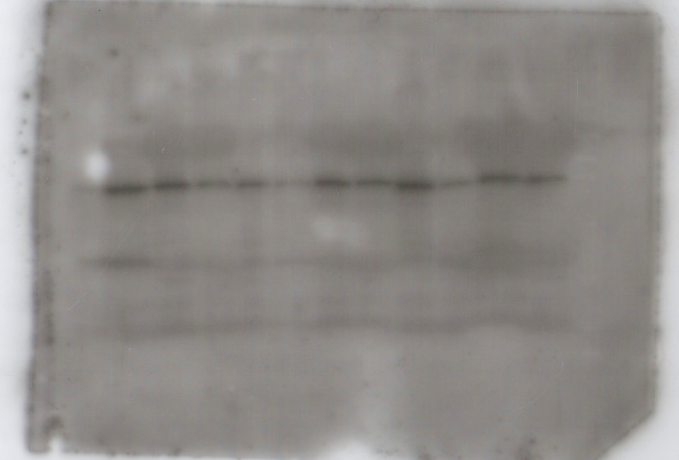


C AEA I A+I C A I A +I

Supplement: Supplementary file 3 — Original Western blot [file 41419_2022_5523_MOESM3_ESM.docx]
